# Broomrape–host interaction: host morphology and physiology as metrics for infestation

**DOI:** 10.1007/s00425-024-04581-1

**Published:** 2024-11-29

**Authors:** Amnon Cochavi

**Affiliations:** Newe Ya’ar Research Center, Institute of Plant Sciences, Agricultural Research Organization - Volcani Institute, 3009500 Ramat Yishay, Israel

**Keywords:** Combined stress, Hydraulic adjustment, Photoprotection, Physiological adaption

## Abstract

**Main conclusion:**

In contrast to other plant pests, broomrape, parasitic plant, rely on maintaining the productivity of the host plant to complete their life cycle.

**Abstract:**

Parasitic plants, particularly those in the *Orobanchaceae* family, rely on their host plants to complete their life cycle. Unlike other plant parasites such as fungi and bacteria, which exploit their hosts regardless of their physiological status, parasitic plants development is linked to the host productivity due to their mutual physiological dependence on water availability and sugar metabolism. Presently, most research focuses on the damage caused to the host after the parasite completes its life cycle, including inflorescence emergence and seed dispersal. However, the interaction between parasite and host begins long before these stages. This implies that certain physiological adaptations are necessary to sustain the parasite’s development while maintaining the host's productivity. In this review, I compile existing knowledge regarding changes in host physiology during the early developmental stages of parasitic plants, spanning from attachment to inflorescence emergence. Additionally, I highlight knowledge gaps that should be addressed to understand how hosts sustain themselves throughout extended periods of parasitism.

## Introduction

In the plant kingdom, obligatory parasites are completely dependent on their hosts’ physiological functioning to complete their life cycles. The relatively long period of parasitism forces the parasite both to exploit the host plant and to ensure host survival until its own reproductive organs mature. In his commentary, Delavault (2020) suggests that the host’s relationship with root parasitic plants is similar to that with other pathogens (insects, bacteria, viruses and fungi) due to the activation in the host of a complex immune system response to the ‘invader’. However, unlike other pests, root parasitic plants and broomrape specifically, require optimal conditions for their development, similar to the needs of their hosts (Hegenauer et al. 2017). Specifically, the physiological functioning of the host plant is crucial for parasite survival.

Parasitic plants are a special group of plants that rely on other living autotrophic plants for nutrition and survival. The parasitic plants attach to the host plant through the root or the shoot by specialized organ named haustoria, which allow them to attract their required water, sugars, and minerals, directly from the host phloem or xylem (Hibberd et al. 1999). Some of the parasitic plants can complete their life cycle with or without the host plant (e.g. *Rhinanthus*) while other can only grow on a host.

Over the years, many works point on the changes induced by the host: changes in the host xylem pressure and nutrient composition (Seel and Jeschke 1999), root structure changes due to modification of the hormonal balance (Spallek et al. 2017), reduced photosynthesis (Frost et al. 1997), and others. Recent years research suggest that small RNA molecules transfer between the parasite and the host can explain the modification or adaption of both the parasite and the host (Shen et al. 2020; Garcia et al. 2021; Mutuku et al. 2021; Clarke et al. 2024).

Among the obligate plant parasites, the Orobanchaceae family represents the largest group. Genera within this family, such as *Orobanche* (broomrape) and *Striga*, primarily parasitize annual plants, particularly crops (Heide-Jørgensen 2013). The two genera differ in their physiological capabilities: most of the *Striga* species are able to assimilate carbon and transpire water in addition to the absorption of essential nutrients from the host plant (Press et al. 1987; Frost et al. 1997), while *Orobanche* and *Phelipanche* species lack photosynthetic components and cannot assimilate carbon independently (Hibberd et al. 1998). Additionally, the impact of *Striga* on host plant carbon assimilation and transpiration is immediate and significant after attachment (Press et al. 1987), whereas the detrimental effects of *Orobanche* and *phelipanche* attachment on host physiology become apparent only after the emergence of the parasite's inflorescence (Pincovici et al. 2018). Thus, the interactions between host plants and *Orobanche* or *Striga* are distinct, shaped by the differing physiology of the parasites.

The importance of these parasitic plants thus lies in their ability to impair yields and even to cause total yield loss (Jacobsohn and Kelman 1980; Bernhard et al. 1998; Hibberd et al. 1999; Eizenberg et al. 2001). Moreover, the parasite infection can lead to a reduction in yield quality in terms of sugar content (Péron et al., 2017; Schaffer et al. 1991), compositions of amino acids (Nandula et al. 2000; Longo et al. 2010; Nativ et al. 2017) and carotenoids (Ortiz-bustos et al. 2017; Emran et al. 2020). Thus, most of the research on broomrapes has been devoted to investigating the effect of the parasite on the host in terms of host productivity and yield quality (Grenz et al. 2008; Hershenhorn et al. 2009; Eizenberg et al. 2012; Cochavi et al. 2015, 2016). Nonetheless, some studies have researched the physiological effect of broomrape parasitism on host functioning. These studies have found that the effect of the parasitism becomes significant only after the parasite has completed its life cycle (Barker et al. 1996; Alcántara et al. 2006; Mauromicale et al. 2008; Longo et al. 2010; Cochavi et al. 2018; Amri et al. 2021; Kalariya et al. 2024). During all the below-ground stages—from the formation of a vascular connection, through tubercle development, up to the formation of the flowering meristem only minor effects on the host plant physiology in terms of morphology and physiology, particularly photosynthesis and hydraulic functioning were described. However, despite these studies, knowledge about the physiological changes occurring in the host plant to accommodate the parasite’s physiological and metabolic demands remained sparse until some years ago. As mentioned before, recent research has demonstrated that RNA, proteins, and other molecules are transferred during the parasite–host interaction (Aly 2013; Moreau et al. 2016; Dubey et al. 2017; Shahid et al. 2018). It has also been observed that the attachment of the parasite can trigger the activation of the host plant's immune system. (Leman et al. 2024; Bouraoui et al. 2024). These studies—most of which focus on the transfer during the formation of vascular connections in the early parasitism stage—suggest that the translocation of molecules allows the parasite to manipulate the host for its own needs. However, the effect of this translocation on the host functioning is not fully understood.

In the current review, I will focus on what is known about adaptions made by the host plant to maintain both itself and its parasite. Or, in other words, this manuscript will review the known changes in the plant morphology and physiology forced by the parasite infection and how they contribute to maintaining the host-parasite interaction until the parasite completes its life cycle. In so doing, it will emphasize the knowledge gaps impeding a comprehensive understanding of the changes forced on the host plant during development of the parasite. In addition, I will seek to delineate the benefits and disadvantages of these adjustments.

### Morphology

**Fig. 1:**
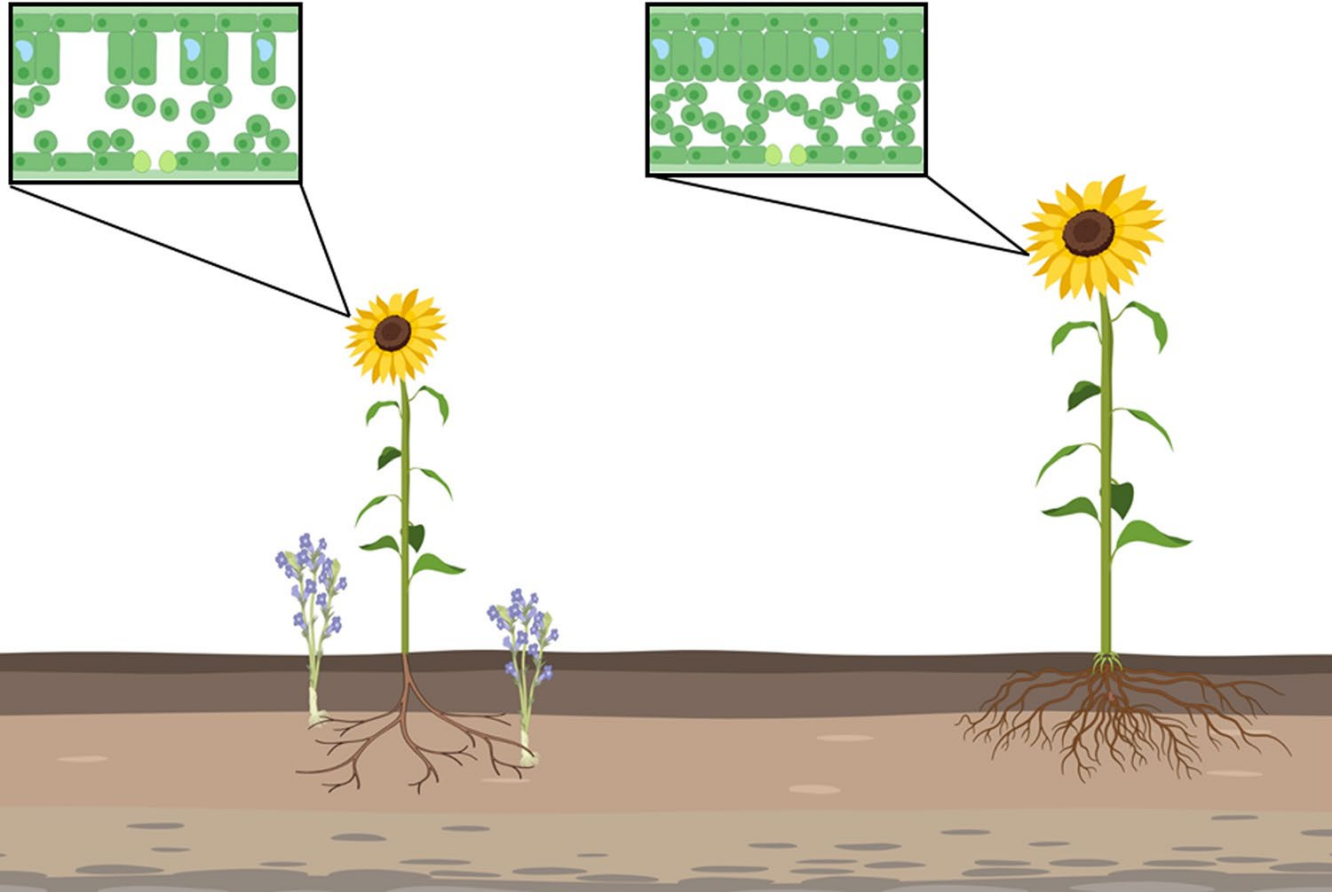
Illustration of the morphological changes in a sunflower plant infested with sunflower broomrape *O. cumana* at the below-ground parasitism stage. As shown, the parasitized sunflower plant has a smaller root system, shorter internodes, and smaller specific leaf area, mainly due to mesophyll loss (these plants also have a lower biomass). The illustration was created using Biorender©

In contrast to the final above-ground stage of the parasite life cycle (emergence of the inflorescence and seed dispersal), the effect of the below-ground parasitism on the host plant is difficult to detect with the naked eye. It is, therefore, necessary to establish metrics for the host plants that indicate broomrape infestations at the below-ground stage, so that estimations of crop damage can be made and suitable control strategies can be implemented (Hershenhorn et al. 2009; Ephrath et al. 2012; Fernández-aparicio et al. 2016). In addition, early detection of the effect of the parasite on the host can contribute to the evaluation of resistant lines for breeding purposes (Ortiz-bustos et al. 2016; Kaundun et al. 2024). Several studies have thus sought to determine the effect of the parasite on the host morphology during the below-ground developmental stage (Fig. 1).

**Fig. 2:**
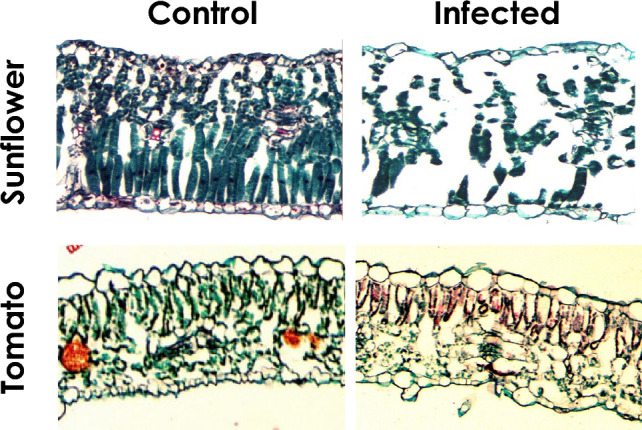
Sections of the youngest fully mature leaf of sunflower and tomato plants after broomrape (*O. Cumana* and *P. aegyptiaca*, respectively) attachment to the host roots. In the sunflower plants, there was a significant difference between control and infected plants in the air space volume. In contrast, in tomato plants there were no differences in leaf mesophyll structure and air spaces. Sunflower images were adapted from Cochavi et al. (2017), and tomato images were taken from Cochavi (2017; unpublished Ph.D. thesis)

Hibberd et al., (1998) demonstrated that infection of sunflower (*Helianthus annuus* L.) with nodding broomrape, *Orobanche cernua*, leads to delayed plant senescence, an increase of leaf area, and a decrease in specific leaf area (g cm^−2^). In their work on the effect of *O. cumana* on sunflower, Pincovici et al., (2018) demonstrated a similar pattern in leaf area and specific leaf area, especially for the first- and the second-youngest leaf pairs. The timing of the parasitism plays a major role in the morphological effect, with early parasitism (immediately after the seedlings emerge) causing a more marked effect than later parasitism. Moreover, removal of the parasite from the host root system (by herbicide application) stops the parasite effect on the leaf pairs that develop thereafter. The decrease in specific leaf area is caused mainly by the reduction in the mesophyll content in the leaf inner area (Cochavi et al. 2017). This reduction appears immediately after the parasite attachment to the host roots and is particular to sunflower plants; for example, in tomato (*Lycopersicon esculentum* Mill.) plants infected with Egyptian broomrape (*Phelipanche aegyptiaca* Pers.), there was no reduction in mesophyll content in the leaves of infected plants (Cochavi 2017 (unpublished); Fig. 2).

In addition to the change in the leaf morphology, the distance between the different shoot segments is also reduced in *O. cumana* infected sunflower plants (Lati et al. 2019). These minor changes in the infected plant can be a result of the formation of a new sink on the root system, or some compensation of the system over the loss of resources; for example, a reduction in host plant biomass may enable the plant to be more efficient in the usage of resources. A reduction in plant biomass, with concomitant maintenance of the carbon assimilating area, enables the host to maintain both itself and its parasite.

### Hydraulic response

Water movement in vascular plants is derived from water potential differences in the plant (Black 1979). The flow of water from the soil through the root and the shoot to the stomata and from there to the atmosphere is controlled by the water potential cascade. Breaking of the water column and the consequent formation of air emboli leads to vessel dysfunction (Ahmad et al. 2018), as can happen when the demand of the leaves for water exceeds the amount that can be supplied by the vascular system. Thus, either high atmospheric demand for water or a low water supply to the vascular system can lead to hydraulic failure (Sperry and Tyree 1988; Tyree and Sperry 1989). The hydraulic response of plants to water limitation may be isohydric, in which stomatal conductance will decrease to prevent unfavorable water and turgor pressure loss, or non-isohydric, which means that the stomata will not respond to the unfavorable water loss so as to maintain a high carbon assimilation rate (Hochberg et al. 2018).

To date, only a limited number of works have investigated the effect of *Orobanche* and *Phelipanche* parasitism on the hydraulic behavior of the host. Among these, some studies showed that the parasite had almost no effect on the transpiration rate in the host plant (Cochavi et al. 2017; Pincovici et al. 2018; Jokinen and Irving 2019). However, the presence of the parasite on the host root system creates a new source that significantly lowers the water potential in the attached root. The parasite's contribution to lowering the water potential involves increasing the osmotic potential through the accumulation of mannitol (Aly 2009; Delavault et al., 2002), and the host’s contribution involves increasing the leaf conductivity to compensate for the creation of a new source. Tomato plants, which tend to have stronger stomatal regulation (isohydric) than sunflower plants, exhibit increased stomatal conductivity when infected by broomrape (Cochavi et al. 2018). In contrast, in sunflower plants, in which stomatal opening is less strongly regulated under normal conditions (non-isohydric), stomatal conductivity does not change upon infection with broomrape, because the conductivity is already maximal. Instead, a reduction in mesophyll tissue reduces the resistance (and therefore increases mesophyll conductivity) of broomrape-infected plants. In addition, the roots' hydraulic conductivity in broomrape-infected sunflower plants is increased, which beside compensates for the reduction in plant surface area and biomass caused by the parasite, can increase its water translocation (Pincovici et al. 2018). This phenomenon is thus opposite to the effect of drought, which leads to a reduction in the plant vessels' conductivity (Savi et al. 2017). Moreover, the reduction in transpiration in broomrape-infected plants is negligible in comparison to the reduction resulting from a water deficit or salt stress (Ashraf & O'Leary 1996; Casadebaig et al. 2008). In infected tomato plants, in which there is a reduction in root biomass (Barker et al. 1996), an increase in stomatal conductance was demonstrated only in the youngest fully matured leaves, but not in the old leaves (Cochavi et al. 2018). However, a combination of broomrape parasitism with salinity stress reduces stomatal conductance. The reason for this difference between infected sunflower (stomatal conductance remains stable under broomrape infection) and tomato plants (increased stomatal conductance) is unclear but it might be the result of the different stomatal responses to water limitation (isohydric vs. non-isohydric), but further research is required.

Increasing the osmotic potential is another way for the plant to reduce the leaf water potential and attract water from the soil (Jones et al. 1980). Increased osmotic potential could be achieved through the accumulation of sugars in the leaves. The connection of broomrape to the host roots is known to reduce the host's carbohydrates concentration in the different plant organs (Whitney 1972; Singh et al. 1972; Schaffer et al. 1991; Cochavi et al. 2018). Also, application of salicylic acid (SA), a plant phytohormone known to mitigate the effect of osmotic stress on the plant (Alavi et al. 2014), on parasitized tomato plants, was found to increase its resistance to broomrape infection through an increase in sugar accumulation (Madany et al. 2020). This study suggests that the parasite’s role as a strong sink influences the host plant's response by altering its sugar balance. To summarize, changes in the hydraulic activity of the host plant in response to broomrape parasitism can affect host carbon assimilation. However, the change in the hydraulic activity (stomatal conductance and transpiration) caused by the parasite is much smaller from the osmotic stress determined by limited water movement from the soil or by other abiotic stressors.

### Photosynthetic activity

Although the broomrape parasitism decreases the availability of water, minerals, and sugar to the host, the photosynthetic activity of the host seems to increase under broomrape infestation (Hibberd et al. 1999). While the carbon assimilation rate remains similar in the youngest and the oldest leaves of both infected and non-infected plants, the intermediate leaves of infected plants exhibit higher photosynthetic activity (Hibberd et al. 1998; Cochavi et al. 2017, 2018; Pincovici et al. 2018). It is known that the presence of a strong sink enables the plant to maintain a high level of carbon assimilation on the leaf level and that removal of the sink will decrease the assimilation rate (Adams et al. 2018). Therefore, the ongoing demand of the parasite for water and nutrients can delay the senescence of the host leaves and increase their carbon assimilation rate. Although the chlorophyll content in the host plant essentially remains the same upon broomrape infection (Longo et al. 2010; Jokinen and Irving 2019), the levels of ribulose-1,5-bisphosphate carboxylase/oxygenase (RuBisCO), the main enzyme of the Calvin Cycle, was found to be higher in infected plants (Hibberd et al. 1999). In that study, despite the enhancement in the photosynthetic activity, the total biomass of the parasite plus infected plants remained similar to the biomass of the non-infected plants. In a different study in tomato plants infected by *P. aegyptiaca*, the combined biomass of the host and parasite plants was found to be lower than that of non-infected plants (Barker et al. 1996). These results suggest that photosynthetic activity by itself is not the limiting factor for biomass accumulation in broomrape-infected plants. Respiration rate, however, may help explain carbon loss, as it has been observed to increase in the root area near broomrape attachment in some infected plants (Singh and Singh 1971).

Several studies have suggested that the limiting factor for host biomass accumulation is the nitrogen deficiency that occurs due the parasite demand (Abbes et al., 2009; Grenz et al. 2008; Jokinen & Irving 2019; Hattori et al. 2024). This notion, in turn, implies that nutrient supplementation can suppress the development of the parasite and reduce the damage to the host (Abu-Irmaileh 1981, 1994; Jain and Foy 1992).

**Fig. 3:**
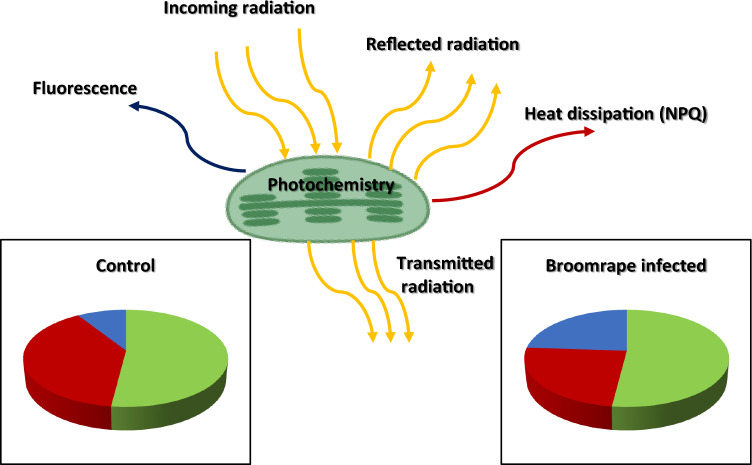
Energy partitioning in the leaf. Incoming photosynthetic active radiation (PAR) can be absorbed by the chloroplasts or reflected/transmitted from the leaf. The absorbed light energy can be used by NADPH and ATP in photosystems (photochemistry; green), dissipated as heat by the plant (NPQ; red) or emitted as fluorescence (blue). The pie charts represent energy partitioning under normal conditions (left panel), and under broomrape infection (right panel) in the absence of any additional stress to the plant

Another aspect of photosynthesis is energy partitioning of the incoming photosynthetic active radiation (PAR) light by the plant. Many studies have demonstrated that in plants under stress there is a tendency to shift absorbed light from the photochemical pathway to the non-photochemical quenching (NPQ) pathway as a means to avoid photoinhibition damage (Shih et al. 2000; Cazzaniga et al. 2013). The NPQ pathway, which serves to shift excess light energy to other energy-demanding pathways (e.g., xanthophyll de-epoxidation in the carotenoid pathway), removes excess energy through heat dissipation (Bilger and Bjorkman 1990; Adams and Demmig-Adams 1994). The marked decrease in carotenoids (and therefore xanthophyll) in broomrape-infected plants, can damage the ability of the host plant to protect itself from excessive light damage to the photosystem (Ortiz-bustos et al. 2017; Emran et al. 2020). Remote sensing of vegetation enables estimation of the xantophyll epoxidation state of plants using the photochemical reflectance index (PRI, Gamon et al. 1992). The exploitation of this idea was demonstrated by Cochavi et al., (2017), who used two distinguish parameters to estimate the regulated non- photochemical quenching level in the host plant under broomrape stress. Both NPQ measured by the Pulse Amplitude Modulation (PAM) technique, and both PRI values measured using proximal spectral device, did not change significantly in *O. cumana* infected sunflower leaves. However, the effect of the parasite on the host has not yet been tested under conditions of high light intensity, which is likely to affect the host response. Under stress conditions, beside the activation of the xanthophyll cycle, there is another energetic pathway to exclude the incoming radiance through emitted fluorescence (Murchie and Lawson 2013; Zait et al. 2024). Rossini et al., (2015) showed that impaired ability of the plant to absorb light through its photosystem leads to higher fluorescence, and Ortiz-bustos et al., (2016) demonstrated that O. *cumana* infected sunflower seedlings emitted a higher fluorescence signal than non-infected control plants. These studies indicate that under a high radiance level the infected plant will use fluorescence as a pathway to dissipate excess energy (Fig. 3). Further research is thus needed to elucidate the effect of the parasite on energy partitioning in the host under high radiance levels.

### Stress combinations

Although the interaction between broomrape and its host occurs under field conditions with some levels of abiotic (and other biotic) stress, most of the relevant studies have investigated broomrape-host interaction physiology under optimal conditions (Hibberd et al. 1998, 1999; Mauromicale et al. 2008; Longo et al. 2010; Cochavi et al. 2017). One such stress is nutrient deficiency, but here, too, the subject has not been exhaustively investigated, although a number of studies have examined different aspects of the effect of nutrient deficiency on broomrape–host interaction physiology. Several studies have focused on investigating the effect of nutrient deficiency on broomrape germination through extraction of hormones (e.g., strigolactone and dehydrocostus lactone) from the root (Joel et al., 2011; López-Ráez et al., 2008; Yoneyama et al., 2012). A small number of works have studied the effect of nutrient deficiency on the host-parasite interaction after establishment of a vascular connection. Jain & Foy, (1992) for example, demonstrated that the addition of nutrients impaired the development of Egyptian broomrape and increased the biomass of host tomato plants in comparison to plant growth in non-infested soil. In contrast, Jokinen & Irving, (2019) showed that although nitrogen supply increased the biomass of host red clover plants, it also increased the biomass of clover broomrape (*Orobanche minor*), such that the biomass ratio remained stable. Specifically, nitrogen application on parasitized or non-parasitized roots of the same plants will encourage the growth of the parasite or the host, respectively (Hattori et al. 2024).

Another important stressor is salinity. It was shown that, while salinity stress reduced the germination and attachment of branched broomrape (*P. ramose*) and *Orobanche* spp. seedlings (Hassan et al. 2010; Demirbas et al. 2013), the attachment of broomrape to the host root increased host sensitivity to salt stress (Cochavi et al. 2018). Due to the lack of soluble sugars, the leaf-level osmotic response to salinity stress that occurs in the rhizosphere is limited. This increased sensitivity to salt pronounced not only in osmotic stress but also with toxification of the plant by chloride and sodium entrance. The reason for the increase in the levels of toxic ions in the parasites plants is still unclear. This phenomenon may result from osmotic adjustments triggered by the presence of the parasite or from other physiological or molecular changes in response to parasitism.

As mentioned earlier, the effect of broomrape parasitism on the host plant involves both photosynthetic and hydraulic systems. However, to date, no work has investigated the effect of high light stress (a light level that leads to photo-damage to the plant) or water deficit on broomrape infected plants. These hydraulic and photosynthetic changes in the presence of the parasite allows to maintain high carbon assimilation and transpiration rates, but they can cause some fitness reduction in the host plant. Future works should fill this knowledge gap to achieve a better understanding of this relation between abiotic stress (drought, high light intensity, etc.,) and broomrape parasitism and their combined effect on the host plant.

## Conclusions

The interaction between broomrape and its host is unique and not similar even to other parasitic plant-plant interactions. Moreover, host plant physiological adaptions are required to ensure the survival of the host plant in order to maintain the parasite. As demonstrated in the current review, the presence of the parasite on the host root forces some physiological and therefore morphological changes in the host plant. Due to the host limited ability to enhance water and nutrient uptake, as well as carbon assimilation rate, to support both its own needs and those of the parasite some morphological adaptions occur. For instance, the host may develop a reduced root system with high hydraulic conductivity to optimize water uptake. Similarly, thinner leaves with a lower specific leaf area help sustain photosynthetic efficiency while minimizing carbon investment. These changes facilitate host plant survival in the face of broomrape parasitism. However, these adjustments probably take some fitness cost from the host and render it susceptible to other biotic and abiotic stressors. This increased vulnerability can subsequently result in amplified cell death and trigger further physiological and morphological alterations in the host plant. Therefore, future works on the parasite-host relationship should take into consideration other factors affecting this relationship. I believe that research under more ‘realistic’ environmental conditions will reveal the real dynamics between the parasite and the host, and the physiological cost of the parasite's presence during its below-ground stage.

## Data Availability

Not relevant.
